# Mobile Apps to Support Caregiver-Resident Communication in Long-Term Care: Systematic Search and Content Analysis

**DOI:** 10.2196/17136

**Published:** 2020-04-08

**Authors:** Rozanne Wilson, Diana Cochrane, Alex Mihailidis, Jeff Small

**Affiliations:** 1 School of Audiology and Speech Sciences Faculty of Medicine The University of British Columbia Vancouver, BC Canada; 2 Department of Occupational Sciences and Occupational Therapy Faculty of Medicine University of Toronto Toronto, ON Canada; 3 Institute of Biomaterials and Biomedical Engineering University of Toronto Toronto, ON Canada; 4 Toronto Rehabilitation Institute Toronto, ON Canada

**Keywords:** mobile apps, communication barrier, dementia, caregivers, long-term care, patient-centered care

## Abstract

**Background:**

In long-term residential care (LTRC), caregivers’ attempts to provide person-centered care can be challenging when assisting residents living with a communication disorder (eg, aphasia) and/or a language-cultural barrier. Mobile communication technology, which includes smartphones and tablets and their software apps, offers an innovative solution for preventing and overcoming communication breakdowns during activities of daily living. There is a need to better understand the availability, relevance, and stability of commercially available communication apps (cApps) that could support person-centered care in the LTRC setting.

**Objective:**

This study aimed to (1) systematically identify and evaluate commercially available cApps that could support person-centered communication (PCC) in LTRC and (2) examine the stability of cApps over 2 years.

**Methods:**

We conducted systematic searches of the Canadian App Store (iPhone Operating System platform) in 2015 and 2017 using predefined search terms. cApps that met the study’s inclusion criteria underwent content review and quality assessment.

**Results:**

Although the 2015 searches identified 519 unique apps, only 27 cApps were eligible for evaluation. The 2015 review identified 2 augmentative and alternative cApps and 2 translation apps as most appropriate for LTRC. Despite a 205% increase (from 199 to 607) in the number of augmentative and alternative communication and translation apps assessed for eligibility in the 2017 review, the top recommended cApps showed suitability for LTRC and marketplace stability.

**Conclusions:**

The recommended existing cApps included some PCC features and demonstrated marketplace longevity. However, cApps that focus on the inclusion of more PCC features may be better suited for use in LTRC, which warrants future development. Furthermore, cApp content and quality would improve by including research evidence and experiential knowledge (eg, nurses and health care aides) to inform app development. cApps offer care staff a tool that could promote social participation and person-centered care.

**International Registered Report Identifier (IRRID):**

RR2-10.2196/10.2196/17136

## Introduction

### Background and Rationale

With the growing aging population, there are more people living with chronic conditions that contribute to physical, sensory (vision/hearing), and cognitive limitations. The complex health care needs of older adults living with chronic conditions may require the services offered in long-term residential care (LTRC) homes. Most LTRC residents (85%) are functionally dependent and require care staff assistance (eg, nurse and residential care aide) while completing activities of daily living (ADLs) [1], and 25% of residents live with dual sensory loss (hearing and vision) [2]. Besides physical and sensory limitations, an estimated 90% of residents live with some cognitive impairment, with 2 out of 3 residents living with Alzheimer disease and related dementias (ADRD) [1]. Furthermore, many residents experience communication difficulties associated with chronic conditions (eg, sensory loss, dementia, and stroke) and/or a cultural-language mismatch with care staff that can challenge interpersonal relationships, and care staffs’ ability to meet residents’ unique needs [3].

Implementation of a person-centered philosophy of care and person-centered interventions in LTRC depends on effective caregiver-resident communication [4]. Person-centered communication (PCC) involves sharing information and decisions between care staff and residents, being compassionate and empowering care provision, and being sensitive to resident needs, preferences, feelings, and life history [5]. By creating an environment that uses strategies and tools to enhance PCC, LTRC care staff can meet residents’ unique needs and foster interpersonal relationships with the residents [6]. For example, care staffs’ use of social and task-focused communication strategies (eg, greet the resident and provide one direction at a time, respectively) with residents living with dementia support the successful completion of ADLs [7,8]. Verbal and nonverbal behaviors (eg, use the resident’s name and make gestures) contribute to positive communication between residents and care staff with different linguistic/cultural backgrounds [3].

Although guidelines for supporting person-centered language in LTRC exist [9], the LTRC setting faces many challenges that can act as barriers to PCC. One such challenge is language diversity. In countries that have a history of welcoming immigrants (eg, Canada, the United States, and Australia), care staff and residents with diverse linguistic and ethnocultural backgrounds often comprise LTRC settings [10-14]. For example, in Canada, most immigrant seniors live in urban areas (eg, Vancouver and Toronto), with approximately 50% of the Vancouver senior population being immigrants [15]. Similarly, it is common to find that English is not the first language of residential care aides, nor are they born in Canada [16]. Therefore, diversity in the LTRC setting is typical in major Canadian urban areas, leading to mismatches between care staff and residents’ first language and/or ethnocultural backgrounds. The shortage of qualified care staff, low wages among residential care aides, and restrictions on who can provide specific types of care can lead to a reduction in the time needed to foster frequent, quality interpersonal interactions with residents [17]. Finally, resource constraints inherent to the LTRC setting (eg, time and staffing) can lead to *task-focused* care rather than *person-focused* care and to fewer instances of caregiver-resident interpersonal interactions [18].

Several traditional approaches to supporting caregiver-resident communication have been tried in LTRC, including professional medical translator services for non-English–speaking residents, communication training programs [19], evidence-based communication strategies [7,8], employing bilingual care staff [20], and using augmentative and alternative communication (AAC) techniques, tools, and strategies (eg, communication boards and gestures). AAC can be used to address the needs of residents living with acquired communication disorders (eg, aphasia and dementia) by supplementing remaining speech abilities or replacing the voice output when speech is no longer viable [20,21]. Although the aforementioned supports can be beneficial, they are often inaccessible to caregivers or residents because of the limited time available for training and/or implementation during care routines, limited funding, and limited on-demand availability.

There is growing recognition of the potential role of technology in supporting the health care of older adults [21], with a focus on person-centered care [22-25]. In particular, the use of mobile communication technology (MCT), which includes mobile devices such as tablets and smartphones, along with their software apps, offers an innovative approach for supporting person-centered care. There are several advantages to using MCT in health care settings: (1) the devices are accessible, portable, small, lightweight, rechargeable, relatively easy to use, and inexpensive, have advanced features (eg, camera and sound recording), and have enough computing power to support web searching; (2) a variety of apps are available in the major app marketplaces; and (3) a wireless connection offers continuous, simultaneous, and interactive communication from any location [26].

In a short period, the availably of mobile apps has increased exponentially across the 2 largest app marketplaces: Google Play (Android platform) and the App Store (iPhone Operating System [iOS] platform). For example, in 2014, there were an estimated 2.6 million apps across the 2 marketplaces [27] and, by 2019, this number climbed to 5.5 million apps (111% increase) [28]. In addition to the convenience and commonplace of MCT, the appeal of using apps in health care may be because of the range of available built-in features that can support individuals’ needs, preferences, and abilities (ie, person-centered care), including larger touch screen interfaces with tactile feedback, motion sensors, voice recognition, cameras, video recorders, and multimedia content (eg, images, sound, and text) [29]. App content can also be customized to support the unique needs of a target population. For example, apps designed for older populations can incorporate larger text and zoom capability; allow for preferred vocabulary, photos, and text; and have the options to save voice and video recordings. Thus, MCTs are useful tools for health care professionals and can support target populations with specific needs, such as those living with ADRD [29-32]. However, more information is needed to determine how these technologies could address specific challenges that caregivers encounter with target populations (eg, dementia [33]) living in LTRC. Furthermore, given the rapidly changing landscape of the app marketplace (eg, new, updated, and removed apps), it is important to better understand the stability of apps in the marketplace. The longevity of apps has important implications in the LTRC context. For example, for training care staff to use an app that is subsequently removed from the marketplace would be a waste of financial resources. The first step to examining the use of MCT in the LTRC setting is to better understand the suitability of currently available commercial apps for supporting communication in the LTRC context and the stability of these apps over time.

Using mobile devices, along with AAC apps and language translation apps, collectively referred to as communication apps (cApps) in this paper, may offer an innovative approach to enhancing PCC in LTRC. In particular, cApps have the potential to support care staff and residents living with acquired neurogenic communication disorders [34] and/or linguistic/ethnocultural barriers [14] during daily activities. For example, cApps could be used as follows: (1) support residents’ participation in their own care; (2) help identify, save, and share residents’ individualized needs and preferences during care routines; (3) personalize activities and social engagement; (4) support information sharing between care staff and residents during daily care; (5) prevent and/or overcome communication breakdowns during ADLs by meeting residents’ unique needs; and (6) promote social participation. However, to date, there appears to be no evidence about the availability of cApps that could support communication between care staff and LTRC residents during daily care routines. Recently, regulations and guidelines for the development and use of technologies in health care have been developed [35]. However, the existing commercially available apps were likely developed with limited regulatory oversight, resulting in little evidence for the validity and reliability of app content and questionable quality [36]. Therefore, we need to better understand the availability and the content quality of currently available cApps. This information will help to determine which cApp could be suitable for supporting caregiver-resident communication in LTRC.

### Research Aims

This app review aimed to systematically identify and examine existing commercially available AAC and translation apps (ie, cApps) that care staff could access to support PCC with LTRC residents during daily activities. The specific objectives of this study were as follows:

1. To systematically identify commercially available apps designed for adults living with a communication impairment (AAC apps) and/or experiencing a language barrier (translation apps).
2. To assess cApp content (description of app characteristics and PCC features), with a focus on suitability and relevance to the LTRC setting.
3. To assess the quality of eligible cApps, with a focus on functionality, ease of use, and customization.
4. To recommend the top existing cApps best suited for supporting caregiver-resident communication during ADLs.
5. To replicate the review to better understand how a rapidly evolving app marketplace may impact the suitability and longevity of cApps in the LTRC setting over a 2-year period.

## Methods

### Identification Phase

#### Search Strategy

**Figure 1:**
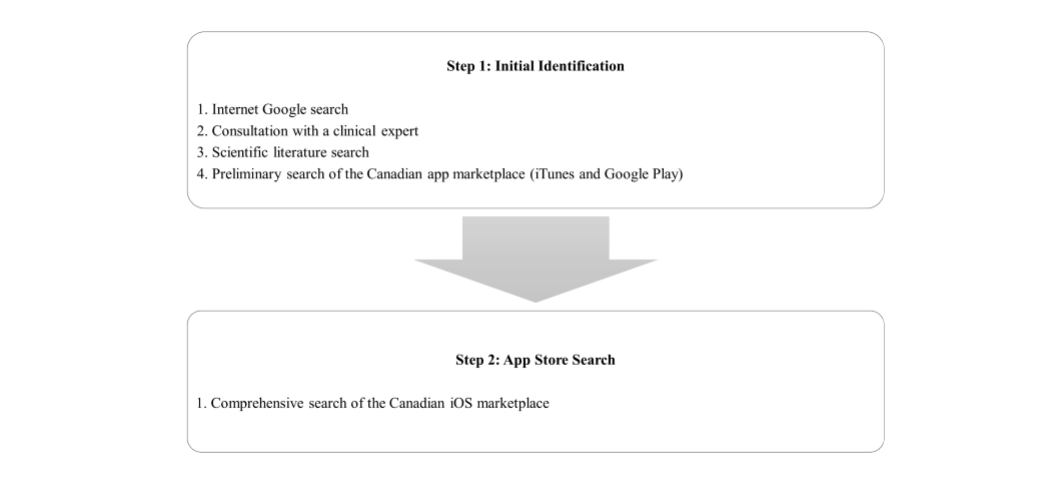
Summary of the steps involved in the identification phase of the communication app reviews. Note that a consultation with a clinical expert and a preliminary search were not conducted for the 2017 review. iOS: iPhone Operating System.

The systematic search for cApps in the Canadian marketplace was conducted between April and June 2015 and involved 5 steps: (1) internet search for AAC and translation apps using the Google search engine; (2) consultation with a speech-language pathologist (SLP) with expertise and knowledge in using AAC apps with adults living with a communication impairment (ie, clinical expert) to identify AAC apps recommended for use by adults living with a communication impairment; (3) scientific literature search focused on the use of mobile apps to support communication in the LTRC setting; (4) preliminary search of the official Canadian app stores of the 2 major operating systems (Android and iOS): Google Play and App Store; and (5) comprehensive search of the Canadian App Store (iOS platform; Figure 1).

#### Initial Identification

To gain a better understanding of the scope of the relevant and/or recommended AAC and translation apps available in the app marketplace, a Google search, a consultation with a clinical expert, a review of the scientific literature, and preliminary marketplace searches were completed. The Google search was conducted to help flag popular AAC and translation apps that should appear in the marketplace searches. The Google searches were done using a Google Chrome web browser by a single author (RW) on the same PC laptop computer (Windows 8; logged into a Google account) and involved separate searches for AAC apps and translation apps (Table 1). Google algorithms place the most relevant search results on the first result page and the majority of searchers stay on the first page [37]. To ensure comprehensiveness, the first 3 pages of the internet search results (50 results per page) were screened for links to specific apps and for links to websites that recommended apps useful for older adults living with a communication impairment or language barrier. Next, a consultation meeting with a clinical expert took place. The SLP shared a detailed spreadsheet of AAC apps that she used with her clients and identified which AAC apps would be appropriate for adults living with a communication impairment in the LTRC setting. The scientific literature search was conducted to identify research reporting on the use of MCT to address the communication needs of vulnerable residents in LTRC. Searches were conducted in MEDLINE, AgeLine, and the Cumulative Index to Nursing and Allied Health Literature academic electronic databases in April 2015 and in February 2016 (RW). Broad search terms were used to capture subdomains of communication challenges/barriers, including language differences or aphasia. Free vocabulary (keywords) and controlled vocabulary (eg, Medical Subject Heading terms) were used for the combined concepts (Table 1). No date restrictions were applied to the searches and search results were limited to peer-reviewed academic literature and the English language. No relevant results were found in the literature searches. Finally, 2 reviewers (authors RW and DC) performed a preliminary search of both the App Store (iOS) and Google Play (Android) on a desktop computer to assess which marketplace appeared to have the highest inventory of AAC and translation apps. On the basis of information gathered from the Google search, the clinical expert, and the preliminary search of both market stores, the App Store (iOS) had the highest inventory of AAC and translation apps.

**Table 1:** Search terms used in Google Chrome and electronic databases.

Search location			Search terms (controlled and free vocabulary)
**Google Chrome**			
	**Function**		
		AAC^a^	augmentative and alternative communication apps for smartphones or tablets
			AAC apps for smartphones or tablet
			adult augmentative and alternative communication apps for smartphones or tablets
			adult AAC apps for smartphones or tablets
			AAC apps for smartphones or tablets for frail elderly
			AAC apps for smartphones or tablets for long-term care residents
			AAC apps for smartphones or tablets in hospital for patients
			communication app for adult patients
		Translation	translation apps for smartphones or tablets
			translation apps for smartphones or tablets
			medical and health care translation apps
**Electronic databases**			
	**Population**		
		Older adults	older adult*, OR *aging* OR *ageing*, OR *aged* OR, senior*, OR elder*, OR frail elder*, OR *dementia*, OR nursing home resident*
			AND
		Caregivers	caregiver*, OR nurse*, OR *nurse aide*, OR health care aide*
			AND
		Communication barrier	*communication*, OR *communication barrier*, OR *communication aids for disabled*, OR *assistive technology*, OR *alternative and augmentative communication*, OR *AAC*, OR *communication disorder*, OR *communication impairment*
			AND
	**Intervention**		
		Mobile communication technology	smartphone*, OR computer*-handheld, OR tablet computer*, OR cell* phone, OR portable computer*, OR mobile app*, OR software app*, OR *computer software*, OR app*
			AND
	**Outcome**		
		Person-centered communication	*Person-centred care*, OR *Personhood*, OR *person-centred communication*, OR *communication strategies*, OR *person-centredness*
			AND
	**Setting**		
		Long-term residential care	*nursing home*, OR *long term care*, OR *institutional care*, OR nursing home patient*, OR *nurse-patient relations*, OR nurse attitude*

^a^AAC: augmentative alternative communication.

#### App Store Search

For this study, the identification of AAC and translation apps that support interpersonal communication between LTRC residents and care staff during ADLs focused on a comprehensive search of the Canadian iOS marketplace: App Store for desktop computer searches and for mobile device searches (tablet and smartphone). AAC apps were searched in the medical, communication, education, lifestyle, and health and fitness categories of the App Store. Several keyword searches were conducted, with the keywords “AAC,” “AAC communication,” “adult communication apps,” and “communication disability” returning most of the search results. The translation apps were searched in the medical education, health and fitness, reference, productivity, utilities, and business categories, using the keywords “translation apps,” “translate apps,” “medical,” and/or “health care translator apps,” and “multi-language translate.” To verify search results, 2 authors (RW and DC) performed independent searches for AAC apps and for translation apps in the App Store, producing similar results.

### Selection Phase

The iOS marketplace search results were exported to Microsoft Excel, and a single reviewer (DC) removed duplicates and screened the remaining apps (names/titles) for being foreign (ie, non-English title) and/or unrelated to interpersonal communication (eg, dictionary app). If the app’s name did not clearly indicate that it was unrelated to communication or was foreign, the app was included for eligibility screening. For apps available in multiple versions, the complete version (ie, fully featured, no limitations, and no in-app purchase required) was included for eligibility screening and the less complete apps were marked as duplicates. In the case of apps with multiple versions that were identical except for the voice setting (male or female), the adult female voice version was selected and the other version was marked as a duplicate. This decision was made because LTRC care staff are typically female [16].

Following the initial screening, the study’s inclusion and exclusion criteria were applied (Textboxes 1 and 2). Two reviewers (authors RW and DC) independently applied the inclusion/exclusion criteria to approximately 20.0% of the apps (AAC: 36/181; translation: 4/18) by reviewing the App Store description. Following acceptable agreement, any disagreements were discussed, with final inclusion/exclusion decisions based on consensus. If needed, a third reviewer (JS) would assist in the inclusion/exclusion decision. A single reviewer (DC) applied the inclusion/exclusion criteria to all remaining apps. AAC apps and translation apps that met all the inclusion criteria, and none of the exclusion criteria, were included for metadata extraction, feature coding, and quality assessment.

**Inclusion criteria for communication apps study eligibility.**

- Communication function: augmentative and alternative communication (AAC)
- The app’s primary function is AAC for adults
- Communication was included as a keyword or in the text description of the app
- Can communicate basic needs (eg, feelings, emotions, preferences, and activities)
- Available in English
- Can support communication between a care provider and a patient in a health care setting
- Can be customized to support individual needs and preferences
- Includes all visual and auditory feedback functions (ie, images, text, and speech/sound)
- Communication function: translation apps
- The app’s primary function is language translation
- Available in multiple languages
- Incudes text-to-speech, speech-to-text, and speech-to-speech translation functions
- Could be used over the web and offline (eg, download language libraries for offline use)
- Option to save words/common phrases to a word bank on a tablet device
- Customization option (eg, save favorite words for quick access)

**Exclusion criteria for communication apps study eligibility.**

- Communication function: augmentative and alternative communication (AAC)
- Requires substantial changes/modifications to use in the long-term residential care setting during the completion of activities of daily living (eg, need to import most images, create text and speech, add/delete built-in features)
- No longer available in the Canadian App Store
- Images are not adult appropriate (eg, child cartoon characters)
- Unrelated to communication with adults living with a communication difficulty
- Unrelated to communicating basic needs
- Does not include all visual and auditory feedback functions (ie, image, text, and speech/sound)
- Communication function: translation apps
- Does not support human-language translation
- Converting English to a single language was the only translation option
- Text-to-text was the only available feature of the app
- No longer available in the Canadian App Store

### Evaluation Phase

#### Data Extraction for Content Analysis

Using a tablet device, a single author (DC) extracted the metadata content for all eligible AAC and translation apps from information provided in the Canadian App Store descriptions. In addition, if available, information was extracted from the developer’s website and/or through reviewing web-based training modules or videos demonstrating the app. For content analysis, the authors (RW and JS) developed a detailed feature coding scheme to guide data collection for each cApp. For each cApp, extracted descriptive data were entered into a standard Microsoft Excel worksheet that contained the following metadata categories: (1) general description: search date, app name, app function, screenshot, keywords, and brief app description; (2) technical information: marketplace/platform, category, language, last software update, cost, and marketplace longevity; (3) target user: general, living with a communication disability, living with aphasia, and other; and (4) other: upgrade with purchase, offline ability, technical support, translation function, and indication of informed design (ie, SLP, clinician, end user, and/or research was used to inform the development of the app).

On the basis of the extracted metadata, a set of secondary selection features were compiled for the AAC apps and for translation apps (Textbox 3). These secondary selection features were considered to be ideal characteristics of an app used in the LTRC setting (eg, a low-cost app with technical support would increase access and offer technical assistance to care staff) and were strongly considered during the identification of the cApps best suited to support PCC during ADLs in the LTRC setting. Evaluated cApps were identified as having a secondary selection feature by indicating *yes* or *no* for the presence or absence of a feature.

**Secondary selection features.**

- Communication function: augmentative and alternative communication (AAC)
- Low cost (app <Can $100 [US $75.5])
- In the marketplace for at least two years (longevity/stability)
- Web-based and offline capabilities
- Technical support (email, phone, and web)
- Includes a translation function
- No cost/low cost for additional languages
- Communication function: translation apps
- Low cost (app <Can $100 [US $75.5])
- In the marketplace for at least two years (longevity/stability)
- Web-based and offline capabilities
- Technical support (email, phone, and web)
- No cost/low cost for additional languages

In addition, during the prepurchase review of the eligible cApps, data were collected on built-in and customizable features that support resident needs, preference, and feeling, as well as sharing of information between residents and care staff (eg, supports vision loss, option to add personal pictures, and two-way communication). All built-in and custom features were coded as being present (*yes*) or absent (*no*) in each app. The detailed feature coding scheme aided in the identification of cApps that included the highest number of PCC features (ie, built-in and customizable) relevant to the LTRC setting.

#### Quality Assessment

For both the AAC and translation apps, quality assessment rating criteria (Table 2) were derived from 3 dimensions of the Mobile Application Rating Scale [38] that were deemed relevant to this study: engagement (customization), functionality (ease of use), and aesthetics (graphic presentation and visual appeal). Each of the criteria was rated on a scale of 0 to 2 (0=*poor*, 1=*fair*, 2=*good* or 0=not at all *easy*, 1=*somewhat easy*, 2=*easy*). cApp quality assessment was conducted in 2 steps: a prepurchase quality assessment and a final quality assessment of purchased cApps. During the prepurchase quality assessment step, 3 reviewers (authors RW, DC, and JS) independently applied the quality assessment rating criteria to the cApps by reviewing the store description, product tutorials/videos, or web-based videos (eg, YouTube) or by downloading freely available cApps. During the prepurchase evaluation, the initial quality assessment did not include ratings on sound quality (AAC) and translation accuracy because this information was typically unavailable without purchasing the app. All apps were assessed in alphabetical order. After ratings were complete, each reviewer judged whether the app was suitable for supporting communication in LTRC (*yes/no/possible*), followed by a decision to purchase/download the app for further evaluation (*yes/no/maybe*).

Following an independent review, the 3 authors convened to comparatively discuss the apps’ initial quality assessment ratings and the apps’ suitability for communication in LTRC. Collectively, the reviewers generated a shortlist of cApps that would be purchased/downloaded to undergo a final quality assessment. Although the cApp ratings were deemed important to the purchase/download decision, cApps that included features appropriate for caregiver-resident communication in LTRC, as well as cApps with customization abilities, were given a higher degree of consideration in the purchase decision. In addition, if there were disagreements between reviewers’ decision to purchase/download a cApp, the undecided cApp was included for further evaluation. Therefore, the approach taken to generate the shortlist could result in the purchase/download of a cApp with a lower median initial quality assessment rating, as well as the decision not to purchase/download a cApp with a higher initial quality assessment rating. Two reviewers (authors DC and JS) independently documented their experience using each shortlisted cApp and completed the final quality assessment for the AAC and translation apps. All shortlisted apps were downloaded to an iPad Mini 4 device with an iOS 9 operating system and a 7.9″ display for a direct user experience.

**Table 2:** Quality assessment rating categories for communication apps.

Communication function^a^	Categories^b,c,d^
Augmentative and alternative communication	Sound quality: *How intelligible is the audio output?* Graphic presentation: *What is the visual interface quality (image resolution detail [pixels] and image clarity)?* Visual interface presentation: *What is the overall appeal of the app look (ie, color display, patterns, lines, scale, image/text type, image/text appropriateness, and display options)?* Ease of use: *Overall, how easy is it to use the software interface (ie, app is intuitive to learn and requires minimal explanation to use; instructions are clear; simple, straightforward display; quick access to common features and commands; well-organized [layout] and easy to navigate)?* Customization: *How easy is it to customize the app?*
Translation	Sound quality Graphic presentation Visual interface presentation Ease of use Translation accuracy: *How accurate are the translated words/text*

^a^The maximum total score for the final quality assessment ratings=10 (augmentative and alternative communication and translation apps).

^b^Sound quality, graphic presentation, visual interface presentation, and translation accuracy were rated on a scale of 0 to 2 (0=poor, 1=fair, 2=good).

^c^Ease of use and customization were rated on a scale of 0 to 2 (0=not at all easy, 1=somewhat easy, 2=easy).

^d^Sound quality and translation accuracy were only applied in the final quality assessment of purchased/downloaded cApps.

### Final Recommendation Phase

Following the independent assessment of all cApps, 3 reviewers (RW, DC, and JS) reconvened to discuss their experience with each app. The final selection of the most suitable cApps in the AAC category and in the language translation category was determined by research team consensus and was based on the combined findings of a three-stage comparative process involving the review of the extracted feature data, the initial quality assessment of eligible cApps, the user experience, and the final quality assessment of the purchased cApps.

### Replication Review

The identification phase of the replication review took place in October 2017, and the evaluation phase was completed in July 2018. Apart from a consultation with a clinical expert, the identification phase involved the same methodological approach as the original 2015 review. Three trained research assistants completed the Google search, the comprehensive iOS marketplace search, and the initial screening (duplicates, foreign, and unrelated), while 1 author (RW) conducted the scientific literature search in October 2017. For the 2017 systematic app review, all search terms used in the Google search, in the comprehensive app store search, and in the literature search were identical to the terms used in the 2015 search (Table 1). As with the 2015 review, Google searches were performed using the Google Chrome web browser and involved separate searches for AAC apps and translation apps. A single research assistant performed all AAC internet searches on the same PC laptop computer, and a single research assistant performed all translation searches on the same PC laptop computer. The literature search returned no relevant results. To replicate the 2015 review, only the Canadian App Store (iOS platform) was searched during the 2017 review.

Two reviewers (RW and DC) completed the selection, evaluation, and recommendation phases of the 2017 replication review. An agreement check was performed for eligibility assessment, whereby 2 reviewers independently assessed approximately 20.0% of the apps (AAC: 61/306; translation: 60/300). Following acceptable agreement, any disagreements were discussed, with final inclusion/exclusion decisions based on consensus and, if needed, a third reviewer (JS). A single reviewer (DC) applied the inclusion/exclusion criteria to all remaining apps. There were two instances in which the procedure for the 2017 replication review differed from the 2015 review. First, multiple versions of the same app (eg, lite [free] and pro [cost]) were treated as unique apps in the 2017 replication review because each version included different features and was anticipated to have varying quality levels. Therefore, a lite version may qualify for evaluation, whereas the pro version may not because of the higher cost. Apps that underwent software updates since the 2015 review were still considered the same version of the app. Second, secondary selection features were applied to eligible cApps before the evaluation phase of the 2017 replication review to further narrow the pool of cApps that underwent quality assessment. Only cApps with all secondary selection features were evaluated in the 2017 review. As with the 2015 review, all quality assessment ratings were completed using an iPad mini 4 (iOS 12.2 operating system and 7.9″ display).

### Data Analysis

Descriptive statistics are used to summarize cApp characteristics, PCC features, and quality assessment ratings. To quantify change between the 2015 review and the 2017 replication review, the number and/or proportional increase/decreases are reported, with numbers/percentages presented from 2015 to 2017 (ie, from *X* to *Y*).

## Results

### Original Review

**Figure 2:**
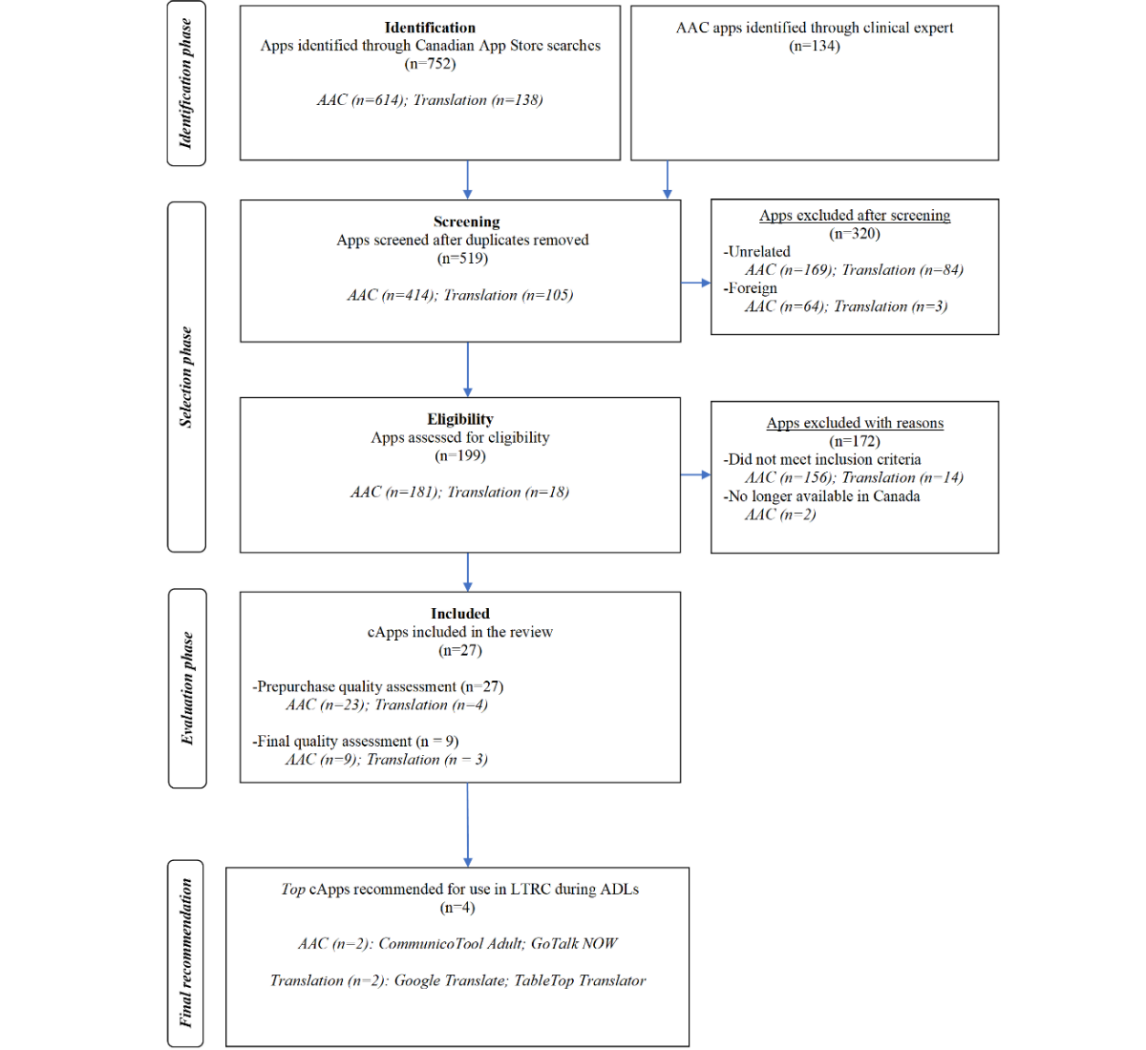
Flow diagram summarizing the results of the identification, selection, evaluation, and final recommendation phases involved in the 2015 communication app review. The presentation of results was guided by the Preferred Reporting Items for Systematic Reviews and Meta-Analyses flow diagram template AAC: augmentative and alternative communication; ADLs: activities of daily living; LTRC: long-term residential care.

The 2015 App Store searches identified a total of 752 cApps (AAC=614; translation=138). The search terms *AAC*, *AAC communication*, and *communication disability* accounted for 90.4% (555/614) of all identified AAC apps. The search terms *translation apps* and *translate apps* accounted for 72.5% (100/138) of all translation apps identified in the initial search. After screening for duplicates, foreign, and unrelated apps, a total of 181 unique AAC apps and a total of 18 unique translation apps were identified. Figure 2 displays the 2015 search results, which was guided by the Preferred Reporting Items for Systematic Reviews and Meta-Analyses flow diagram template [39]. After applying this study’s inclusion/exclusion criteria to the identified apps, 27 cApps were included in the study (Tables 3 and 4).

**Table 3:** List of communication apps evaluated in the 2015 review.

Category	Apps
Augmentative and alternative communication (n=23)	Alexicom Elements Adult Home (Female)^a^ App2Speak^a^^,^^b^ AutisMate365^c^ ChatAble^a^ CommunicAide CommunicoTool Adult^a,d^ Compass (DynaVox) Conversation Coach^a,b^^,^^d^ Easy Speak—AAC^c^ Functional Communication System^c^ GoTalk NOW^a,d^ iAssist Communicator^c^ iCommunicate^a^ image2talkb^b,e^ MyTalkTools^a,d^ PictureCanTalk^a,^^c^ Proloquo2Go^a^ Smart_AAC (med)^d^ Sono Flex^a,d^ SoundingBoard Talkforme^c^ TalkTablet^a,d^ TouchChat AAC
Translation (n=4)	Google Translate^a,b^ iTranslate^a,d^ SayHi Translate^c^ TableTop Translator^a,b^

^a^Indicates that this app met study eligibility in the 2015 review and in the 2017 review.

^b^Indicates that the same version of the app was evaluated in the 2015 and in the 2017 reviews.

^c^Indicates that this app was no longer available in the marketplace during the 2017 review.

^d^Indicates that a different version of the same app was evaluated in the 2017 review (eg, 2015: CommunicoTool Adult; 2017 CommunicoTool 2).

^e^For cApps with multiple versions, if a version of the cApp was evaluated in both the 2015 and in the 2017 review (eg, GoTalk NOW LITE and GoTalk Start different versions [ie, fewer features] of GoTalk NOW), it was not categorized as a newly evaluated cApp.

**Table 4:** List of communication apps evaluated in the 2017 replication review.

Category	Apps
Augmentative and alternative communication (n=25)^a^	App2Speak CommunicoTool 2 Conversation Coach Conversation Coach Lite^b^ CoughDrop^c^ Gabby^c^ GoTalk NOW LITE^b^ GoTalk Start^b^ image2talk iMyVoice Lite^b,c^ iMyVoice Symbolstix^c^ iSpeakUp^c^ iSpeakUp Free^b,c^ LetMeTalk^c^ Mighty AAC^c^ MyTalkTools Mobile Lite SmallTalk Aphasia—Female^c^ Sono Flex Lite^b^ TAlkTablet CA AAC/Speech for aphasia^d^ TalkTablet LITE—Eval Version^b^ TalkTablet US AAC/Speech for aphasia^d^ urVoice AAC—Text to speech with type and talk^c^ Visual Express^c^ Visual Talker^c^ Voice4u AAC^c^
Translation (n=17)	Google Translate Instant Translator—Converse^c^ iTranslate Translator^c^ iTranslator—Speech translation^c^ iVoice^c^ LINGOPAL 44^c^ Microsoft Translator^c^ Multi Translate Voice^c^ Online—Translator.com^c^ Voice Translator Reverso^c^ Speak & Translate—Translator^c^ TableTop Translator The Interpreter—translator^c^ Translator with Speech HD^c^ Translator—Speak & Translate^c^ TravTalk—Talking & Recording Phrasebook^c^ Yandex.Translate: 94 languages^c^

^a^For cApps with multiple versions, if a version of the cApp was evaluated in both the 2015 and in the 2017 review (eg, GoTalk NOW LITE and GoTalk Start different versions [ie, fewer features] of GoTalk NOW), it was not categorized as a newly evaluated cApp.

^b^cApps that were newly evaluated in the 2017 review.

^c^A free or low-cost version of a fully featured app that is available for a higher cost.

^d^A different version of the same app.

#### Content Analysis

Extracted metadata for the evaluated cApps indicated that 91% (21/23) of AAC apps were only available for the iOS platform, while 50% (2/4) of translation apps were available for both the iOS and the Android marketplaces. The majority of AAC apps (18/23, 78%) were categorized as *education* apps and most likely included one or more of the following keywords: AAC (18/23, 78%), communication disability (10/23, 43%), basic needs (4/23, 17%), or daily living (4/23, 17%). Half of the translation apps (2/4) were categorized as *business* apps and all were labelled with the keyword *translate*. The majority of AAC apps (18/23, 78%) identified people living with a communication disability as the target user, whereas all translation apps (n=4) were designed for a general audience. Most AAC apps were available in English only (17/23, 74%), and the last software update was within 1 year (17/23, 74%). All translation apps’ software was updated in the current year (ie, 2015). Some of the free AAC apps were limited versions of an app that could be upgraded with a purchase (eg, *CommunicAide (free)* and *CommunicAide Pro*, Can $99.99 [US $75.5]), and the majority (14/23, 61%) of AAC apps provided no indication of informed design. Only 17% (4/23) of the AAC App Store description and/or the developer’s webpage indicated the inclusion of an SLP in the development of the app (*App2Speak, Chatable, CommunicAide Free,* and *CommunicoTool Adult*), while 9% (2/23) indicated research was used to inform the content (*Compass [DynaVox]* and *Proloquo2Go*), and 13% (3/23) included the end user (*Talkforme, image2talk,* and *MyTalkTools*).

Most of the AAC apps cost Can $100 (US $75.5) or less (17/23, 75%), were available in the marketplace for 2 years or more (18/23, 78%), and provided technical support (22/23, 96%; Table 5). All translation apps cost less than Can $25 (US $18.9), 3 out of the 4 apps provided technical support, and the majority (3/4, 75%) were available in the marketplace for 2 years or more. Although about half of the AAC apps indicated some offline functionality, only 1 translation app (*Google Translate*) had limited offline functionality. Although no AAC app included all secondary selection features, 83% (19/23) of the AAC apps contained three or more of these features. *GoTalk NOW*, *SoundingBoard, AutisMate365, Conversation Coach, Functional Communication System*, and *MyTalkTools* contained the most of these features. Except for online and offline capabilities, 75% (3/4) of the translation apps included each of the secondary selection features.

Appraisal of PCC features indicated that 3 AAC apps (*GoTalk NOW, Talkforme,* and *MyTalkTools*) contained 11 or more of the built-in and custom features. Only 1 AAC app contained all 5 custom features (*GoTalk NOW*) and 1 AAC app included nearly all the built-in features (*Talkforme*). One translation app contained 86% (6/7) of all applicable features (*Google Translate*). Almost half of the AAC apps included 50% to 74% of the features that were deemed to support PCC, and 75% (3/4) of the translation apps contained some of the features (Table 6). Although all AAC apps indicated that they supported hearing loss (eg, speech rate adjustment, voice customization, and speech-to-text function), only 43% (10/23) supported vision loss (eg, high-resolution images, zoom function, and large images) and two-way communication (ie, conversation/interpersonal). All translation apps supported hearing and vision loss. Only 3 AAC apps included a built-in translation function (*Talkforme, MyTalkTools,* and *TouchChat AAC*). The majority of AAC apps included multiple display modes, natural voice output, and text-to-speech output. The ability to add personal photos/images and the option to add personal voice recordings were the most common custom features among the AAC apps, and all translation apps included vocabulary customization.

**Table 5:** Secondary selection feature summary of the evaluated communication apps (cApps; note: as the percentages were rounded, some categories may not add up to 100%.

Secondary feature		2015 review		2017 replication review		Change over time, (%)^d^	
		AAC^a^ (n=23), n (%)	Translation (n=4), n (%)	AAC (n=25)^b^, n (%)	Translation (n=17)^c^, n (%)	AAC	Translation
**Cost in Can $ (low cost: app <Can $100 [US $75.5])**							
	Free	4 (17)	2 (50)	10 (40)	15 (88)	135	76
	<$25 (US $18.9)	2 (9)	2 (50)	6 (24)	2 (12)	167	−76
	$25-$49 (US $18.9-US $37)	7 (30)	0 (0)	4 (16)	0 (0)	−47	0
	$50-$75 (US $18.9-US $37.8)	1 (4)	0 (0)	0 (0)	0 (0)	−100	0
	$75-$100 (US $37.8-US $75.5)	3 (13)	0 (0)	1 (4)	0 (0)	−69	0
	>$100 (US $75.5)	6 (26)	0 (0)	4 (16)	0 (0)	−38	0
In the marketplace for at least two years (longevity/stability)^e^		18 (78)	3 (75)	19 (76)	13 (76)	−3	1
Web and offline capabilities^f^		13 (57)	1 (25)	25 (100)	17 (100)	75	300
Technical support (email, phone, web)		22 (96)	3 (75)	25 (100)	16 (94)	4	25
Includes a translation function		3 (13)	N/A^g^	1 (4)	N/A	−69	N/A
No cost/low cost for additional languages		N/A	4 (100)	N/A	17 (100)	N/A	0

^a^AAC: augmentative and alternative communication.

^b^In total, 11 AAC apps were evaluated in both the 2015 review and in the 2017 review.

^c^In total, 3 translation apps were evaluated in the 2015 review and in the 2017 review.

^d^A negative percentage indicates a decrease in the percentage of cApps with the secondary feature over the 2-year period.

^e^The app copyright date was used to document marketplace longevity. In the absence of a copyright date, the oldest software update date was used.

^f^Functions/features available offline may be limited compared with the features available during app use over the web.

^g^Not applicable.

**Table 6:** Summary of features that support person-centered communication (PCC) in the evaluated communication apps (cApps; this table describes PCC features found in the evaluated cApps during the 2015 review and in the 2017 replication review, as well as evaluates the percentage of cApps with a PCC feature between the 2015 and 2017 reviews. Data were extracted from the Canadian App Store description and during the prepurchase review of the cApps. Feature categories were not mutually exclusive; therefore, 1 app could have several built-in features and/or custom features).

Person-centered communication features		2015 review		2017 review			Change over time, (%)^b^		
		AAC^a^ (n=23), n (%)	Translation (n=4), n (%)	AAC (n=25), n (%)	Translation (n=17)	AAC			Translation
**Built-in features (n=9)**									
	Supports vision loss^c^	10 (43)	2 (50)	8 (32)	9 (53)	−26		6	
	Supports hearing loss^d^	23 (100)	4 (100)	25 (100)	16 (94)	0		−6	
	Multiple display representations^e^	20 (87)	4 (100)	25 (100)	11 (65)	15		−35	
	Natural sounding voice output^f^	16 (70)	3 (75)	17 (68)	11 (24)	−3		−68	
	Text-to-speech function	22 (96)	N/A^g^	25 (100)	N/A	4		N/A	
	Speech-to-speech function	2 (9)	N/A	1 (4)	N/A	−56		N/A	
	Translation function	3 (13)	N/A	1 (4)	N/A	−69		N/A	
	Available in multiple languages	8 (35)	N/A	11 (44)	N/A	26		N/A	
	Supports two-way communication^h^	10 (43)	3 (75)	7 (28)	7 (41)	−35		−45	
**Custom features (n=5)**									
	Can customize vocabulary^i^	7 (30)	4 (100)	17 (68)	12 (71)	127		−29	
	Can add/save personalized photos/images	18 (78)	1 (25)	19 (76)	3 (18)	−3		−28	
	Can to add/save personalized text	8 (35)	N/A	8 (32)	N/A	−9		N/A	
	Option to add/save personal voice recordings	15 (65)	N/A	15 (60)	N/A	−8		N/A	
	Can add/save personalized videos	4 (17)	N/A	6 (24)	N/A	41		N/A	
**Total number of features** ^j^									
	cApps^k^ with all applicable features	0 (0)	0 (0)	0 (0)	0 (0)	N/A		N/A	
	cApps with most features (approximately 75% or more)	3 (13)	1 (25)	2 (8)	3 (18)	−38		−28	
	cApps with some features (approximately 50%-74%)	11 (48)	3 (75)	12 (48)	8 (47)	0		−37	
	cApps with few features (less than 50%)	9 (39)	0 (0)	11 (44)	6 (35)	13		N/A	

^a^AAC: augmentative and alternative communication.

^b^Percent change calculation: ([percentage of 2017 apps with the feature−percentage of 2015 apps with the feature]/percent of 2015 apps with the feature)*100. A negative percentage indicates a decrease in the percentage of cApps with the person-centered communication feature over the 2-year period.

^c^Features that support vision loss include high-resolution images, zoom function, and large pictures/text.

^d^Features that support hearing loss include volume control, earbud option, speech rate adjustment, voice customizations, and speech-to-text function.

^e^Multiple display representations indicate that the app includes two or more features: text, handwriting option, speech input, camera/photo pictures, images, symbols, and video.

^f^Information about voice output was not available for 4 AAC apps during the data extraction phase.

^g^Not applicable.

^h^Supports two-way communication means that the app could be used for caregiver-resident task-focused and/or interpersonal-focused communication (eg, conversation view).

^i^Option to customize vocabulary includes saving frequently used words/phrases in the following manner: pages, favorite lists, history, and add personalized vocabulary.

^j^A total of 14 person-centered features applied to AAC apps (built-in=9; custom=5). A total of 7 person-centered features were applicable for translation apps (built-in=5; custom=2).

^k^cApp: communication app.

#### Quality Assessment

Initial quality assessment of the eligible cApps indicated that 7 AAC apps were highly rated: *Alexicom Elements Adult* (median 8)*, TalkforMe* (median 8)*, App2Speak* (median 7), *CommunicAide* (median 7), *CommunicoTool Adult* (median 6), *Functional Communication System* (median 6), and *GoTalk NOW* (median 6). After considering secondary selection features and the initial quality assessment during a research team discussion, 9 AAC apps and 3 translations apps were shortlisted for purchase/download (Table 7). Following completion of the final quality assessment ratings for each of the shortlisted cApps, *CommunicoTool* *Adult* and *GoTalk* *NOW* had the highest median ratings for AAC apps (Table 7). The research team reconvened for a final comparative review of the cApps. On the basis of consensus decisions, the *top* recommended cApps were finalized: *CommunicoTool Adult, GoTalk NOW, Google Translate,* and *TableTop Translator* (Multimedia Appendix 1). Although *TableTop Translator and SayHi* shared the same developer, the researchers selected *TableTop* *Translator* because this app included more language options and the screen display supported two-way communication.

**Table 7:** Communication apps downloaded for final quality assessment ratings (the final quality assessment rating is based on the median rating of 2 reviewers. The maximum total rating score for cApps apps was 10).

cApp communication function and name^a^			Quality assessment ratings		
			2015 search (n=12)		2017 search (n=8)
**Augmentative and alternative communication**					
	App2Speak	6.5		8.5	
	CommunicoTool Adult	9^b^		8.5^b,c^	
	Functional Communication System	5		N/A^d^	
	GoTalk NOW	7.5^b^		8^b,e^	
	iAssist Communicator	0		N/A	
	iCommunicate	3		N/A	
	image2talk	0		N/A	
	SoundingBoard	1		N/A	
	Talkforme	1.5		N/A	
**Translation**					
	Google Translate	8^b^		9.5^b^	
	iVoice Translator	N/A		9.5^b^	
	Microsoft Translator	N/A		10^b^	
	Online-Translator.com	N/A		8	
	TableTop Translator^f^	8^b^		8.5	
	SayHi Translate	8		N/A	

^a^Communication apps (cApps) are listed in alphabetical order.

^b^Top recommended cApps for use in long-term residential care to support communication between residents and caregivers.

^c^*CommunicoTool 2* was evaluated in the 2017 review.

^d^Not applicable.

^e^GoTalk NOW LITE was evaluated during the 2017 review.

^f^*TableTop Translator* and *SayHi* shared the same developer.

### Replication Review

#### Content Analysis

**Figure 3:**
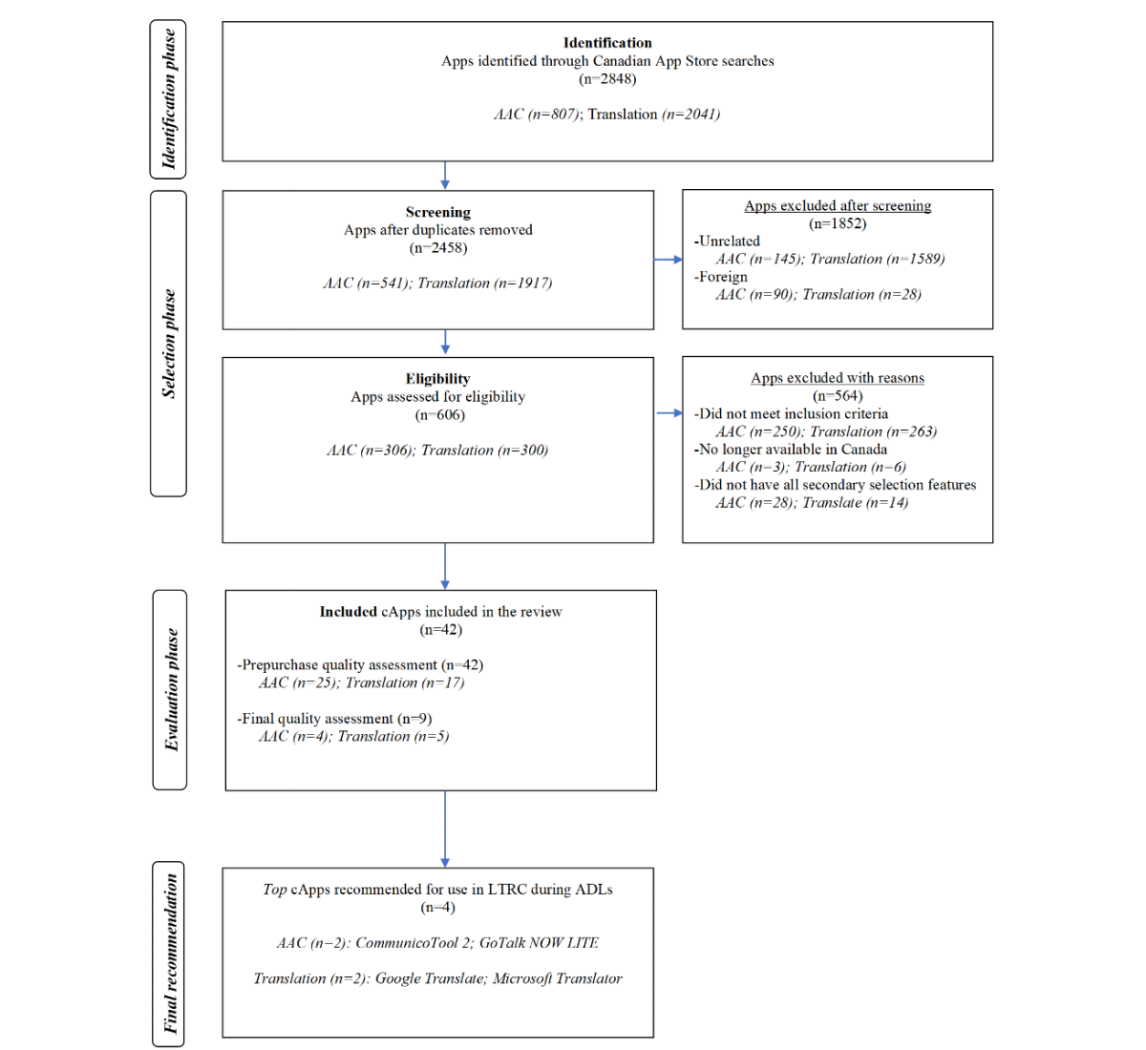
Flow diagram summarizing the results of the identification, selection, evaluation, and final recommendation phases involved in the 2017 communication app replication review. The presentation of results was guided by the Preferred Reporting Items for Systematic Reviews and Meta-Analyses flow diagram template AAC: augmentative and alternative communication; ADLs: activities of daily living; LTRC: long-term residential care.

Following a comprehensive search of the App Store and the removal of duplicates, foreign, and unrelated apps, a total of 607 apps were screened for study eligibility (Figure 3). A total of 93 apps met the study’s inclusion criteria. After applying the secondary selection features to further narrow down the pool of cApps, a total of 42 apps were evaluated (AAC: n=25; translation: n=17; Tables 3 and 4). In all, 36% (9/25) of the evaluated AAC apps were a different version of the same app (eg, *Conversation Coach* and *Conversation Coach Lite)*. A total of 28% (7/25) of the evaluated AAC apps were a low-cost or free version of an app that was also available in a fully featured version for a greater cost (Tables 3 and 4). None of the evaluated translation apps was a different version of the same app. The majority of the AAC apps were available only for the iOS platform (19/25, 76%), cost less than Can $25 (US $18.9) or were free (16/25, 64%), and were only available in English (14/25, 56%). Only 3 AAC apps indicated informed design (SLP: *Apps2Speak* and *Voice4u AAC*; end user: *image2talk*), and only 1 AAC app included a translation function (*LetMeTalk*). Most translation apps were only available in the iOS marketplace (15/17, 88%), were available for 2 years or longer (13/17, 76%), were free (15/17, 88%), and offered technical support (16/17, 94%). All translation apps had recent software updates and had some offline functions (Table 5).

The majority of cApps contained at least some PCC features (AAC: 14/25, 56%; translation: 11/17, 65%; Table 6). The AAC apps with the highest number of PCC features were *GoTalk NOW LITE* (11/14), *GoTalk Start* (11/14)*,* and *CommunicoTool 2* (10/14). The translation apps with the highest number of PCC features were *Google Translate* (6/7), *TableTop* *Translator* (6/7)*, Translator with Speech HD* (6/7*), Microsoft Translator* (5/7)*, Multi Translate Voice: Say It* (5/7)*, and Voice Translator Reverso* (5/7)*.* All AAC apps supported hearing loss, included multiple display representations, multiple output modes, and a text-to-speech function, while very few included a speech-to-speech function or a translation function.

#### Quality Assessment

All cApps that underwent final quality assessment were highly rated (Table 7). On the basis of researcher consensus, the following cApps were deemed to be best suited for supporting communication between residents living in LTRC and their caregivers during ADLs: *GoTalk NOW LITE, CommunicoTool 2, Google Translate,* and *Microsoft Translate* (Multimedia Appendix 1). Although *App2Speak* was rated higher than *GoTalk NOW LITE*, the app contained fewer PCC features than *GoTalk NOW LITE* (8 and 11, respectively) and fewer features than *CommunicoTool 2*. Importantly, *App2Speak* included only 2 custom features (add personal pictures and voice recordings) compared with *GoTalk NOW LITE*, which contained 4 custom features.

#### Stability of Evaluated Communication Apps Over Time

Between the 2015 review and the 2017 replication review, the number of AAC apps identified in the iOS marketplace increased by 31.4% (from 614 to 807) and the number of identified translation apps increased exponentially (from 138 to 2041; Figures 2 and 3). In all, 61% (14/23) of the eligible AAC apps in the 2015 review also met study eligibility in the 2017 review (Tables 3 and 4). Of the 2015 eligible/evaluated AAC apps, 35% (8/23) were no longer available in the marketplace in 2017. Two AAC apps identified in the 2015 review were excluded in the 2017 review because one required a substantial change for use in the LTRC setting and the other included images that were not adult appropriate (eg, child cartoon images; *SoundingBoard and TouchChat AAC*, respectively). Finally, 1 AAC app was excluded from the 2015 review but was deemed eligible in the 2017 review (*Gabby)*. The images in *Gabby* were categorized as not adult appropriate in the original 2015 review; however, the updated version of *Gabby* contained images that were considered adult appropriate. Except for the *SayHi* app, which is no longer available in the App Store, all translation apps that were eligible in the 2015 review also met study inclusion in the 2017 review.

A similar number of AAC apps were evaluated in the 2015 and the 2017 reviews (n=23 and n=25, respectively). Of the 25 AAC apps evaluated in the 2017 review, 3 (12%) were the same version of the app evaluated in the 2015 review (*App2Speak, Conversation Coach,* and *image2talk*) and 9 (36%) were a different version of the same app evaluated in the 2015 review (eg, *Conversation Coach Lite* and *Sono Flex Lite*; Tables 3 and 4). Notably, *CommunicoTool Adult* (2015 review) was no longer available in the App Store and was replaced by *CommunicoTool 2* (2017 review). For this study, *CommunicoTool 2* was considered a different version of the same app because its features were quite like those of *CommunicoTool Adult*. Although *GoTalk NOW* was the only version of the app that was evaluated in the 2015 review, *GoTalk NOW, GoTalk NOW PLUS*, *GoTalk NOW LITE*, and *GoTalk Start* were all eligible versions of the same app in the 2017 review. After applying the secondary selection criteria, only *GoTalk NOW LITE* and *GoTalk Start* were evaluated in the 2017 review because these versions were classified as low cost (<Can $100 [US $75.5]). The cost of *GoTalk NOW* increased by 22% (from Can $89.99 [US $67.9] to Can $109.99 [US $83.1]). The number of evaluated translation apps increased by 325% in the 2017 review (from 4 to 17). Three of the four translation apps (75%) evaluated in the 2015 review were also evaluated in the 2017 review.

#### Stability of Communication App Features Over Time

Over the 2-year period, the majority of the evaluated cApps were only available for the iOS marketplace; however, the largest increase was observed in the percentage of AAC apps available across both iOS and Android platforms (167%; from 2/23, 9% to 6/25, 24%), and a decrease occurred among the translation apps (76%; from 2/4, 50% to 2/12, 12%). Between 2015 and 2017, the majority of AAC apps continued to indicate that adults living with a communication disability were the target user, while translation apps continued to target the general users. There was a 44% increase in the percentage of AAC apps with no indication of informed design (from 14/23, 61% to 22/25, 88%), and the largest percent decrease was seen in AAC apps that included a translation function (69%; from 2/23, 13% to 1/25, 4%). For the secondary selection features (Table 5), only two remained stable over time across cApps: in the marketplace for 2 years or more and available technical support. The largest percent change increase was observed in AAC apps that were free (135%; from 4/23, 17% to 10/25, 40%) or cost less than Can $25 (US $18.9; 167%; from 2/23, 9% to 6/25, 24%) and in translation apps that included web-based and offline capability (300%; from 1/4, 25% to 17/17, 100%).

For PCC features, the overall percentage of AAC apps that included approximately 50% to 74% of the PCC features remained stable over the 2-year period (48%), whereas the percentage of evaluated translation apps with at least some PCC features decreased by 37% between 2015 and 2017 (from 3/4, 75% to 8/17, 47%; Table 5). Many of the custom PCC features included in AAC apps remained stable over the 2-year period, specifically features that supported hearing loss, used a natural sounding voice output, included a text-to-speech function, and offered an option to add/save personalized photos/images. Between 2015 and 2017, the largest percent increase occurred among AAC apps that included an option to customize vocabulary (127%; from 7/23, 30% to 17/25, 68%), whereas the largest decrease occurred for the percentage of AAC apps that included a translation option. Over the 2-year period, translation apps witnessed the largest decrease among the percentage of apps that included a natural sounding voice (68%; from 3/4, 75% to 11/25, 24%), whereas the percentage of translation apps that supported two-way communication decreased by 45% (from 3/4, 75% to 7/25, 41%).

## Discussion

### Principal Findings

This study’s comprehensive review of cApps available in the iOS marketplace aimed to identify and assess the features and quality of cApps that would be most appropriate for use with residents living in LTRC homes. In addition, this study examined the stability/instability of cApps over a 2-year period. The 2015 review process culminated in selecting 2 AAC apps (*CommunicoTool Adult and GoTalk NOW*) and 2 language translation apps (*Google Translate and TableTop Translate*) that provided the most suitable overall content and usability features for enhancing communication between care staff and residents living in LTRC. For purposes of augmenting communication with images, video, sound, and text, these top 2 AAC apps contained features and functionality that promote a multimodal understanding of messages, appealing and high-quality images and audio/video capabilities, and the capacity to customize content to individuals. One of these AAC cApps, *GoTalk NOW*, has received an endorsement from researchers in the field of AAC [40]. The top 2 language translation apps in the 2015 review offered features that provided high-quality voices, accurate translation, the capacity to save commonly translated phrases, and versatility in translating across modalities (eg, text to speech). Together, these 4 cApps provide a promising starting point for integrating communication technology into LTRC person-centered care practices. It is interesting to note that, during the predownload initial quality assessment, the top recommended cApps did not have the highest median quality assessment ratings. For example, *Alexicom Elements Adult, Talkforme, App2Speak,* and *CommunicAide* received the highest median rating for AAC apps, and *SayHi Translate* was the highest-rated translation app. However, once the shortlisted cApps were downloaded and used, the respective features, functionality, and usability of the top recommended cApps were judged to be superior to all the other downloaded apps. For example, *CommunicoTool Adult* included the option to have a human voice, the built-in photos were clear and relevant, and the app was customizable, and *GoTalk NOW* was easy to use, had several built-in and customizable features, and the stock pictures were relevant. *Google Translate* allowed for web-based and offline (ie, saved phrases) functions, was free, and was easy to use, whereas *TableTop Translator* supported face-to-face conversation with a unique split-screen function.

Overall, the majority of cApps evaluated in 2015 (20/27, 74%) demonstrated marketplace stability over a 2-year period. In the 2017 review, only one of the top recommended cApps from the 2015 review was replaced with a newly evaluated translation app, whereas the top AAC apps were different versions of the same app recommended in the 2015 review. The decision to recommend *Microsoft Translator* over *TableTop Translator* was based on several factors. The visual interface quality, the sound quality, and the visual interface presentation of *Microsoft Translator* were rated higher compared with *TableTop Translator*. Also, *TableTop Translator* uses Microsoft for translations, had not undergone any recent updates, and the app crashed several times while attempting to translate when using the app. Although *CommunicoTool Adult* was replaced by *CommunicoTool 2*, the newer version remained a top recommended cApp for use in the LTRC setting to support caregiver-resident communication during ADLs.

Although many of the AAC apps evaluated in 2015 and in 2017 include features and functionality that could support communication between LTRC staff and residents (ie, support hearing loss, included multiple display options, a text-to-speech function, add personal photos; technical support), less than half of AAC apps contained some (ie, 50%-74%) of these features. For instance, in both reviews, there was a limited number of evaluated AAC apps that supported two-way communication, included a speech-to-speech option or a translation function, supported vision loss, or provided options to add/save personalized text or videos. Moreover, the majority of AAC apps provided no indication of informed design, with less than 10% indicating SLP involvement in the design/development of the app. Importantly, it appears that none of the cApps, including the ones shortlisted in the 2015 and 2017 reviews, were specifically developed to support PCC, particularly with frail elderly residents living with sensory, motor, or cognitive impairments, and/or language barriers. For example, the stored voices linked to images in AAC apps (eg, speaking the word *orange* when clicking on image of orange) and translator’s voices have not taken into account the potential impact of speaker/listener dialect or accent, nor the use of male versus female voice, on residents’ and staff’s ability to understand the voice. The images on these apps are also generic, which means that some of the images are not relevant for the LTRC context because they have a different appearance than what is encountered in the resident’s specific care environment (eg, dining area, shower, and meals or snacks). Using voices from the same dialect of the residents with voice qualities that accommodate to the high-frequency hearing loss of many residents, along with images that align with elderly residents’ current and previous life experiences, is an important way to reduce the information processing demands of residents and maximize their familiarity with the content. In view of older adults’ reluctance to learn new technologies, making the content as relevant and meaningful to their life experience and current needs should promote person-centered care and, thereby, greater acceptance of MCT and cApps during their daily activities.

All AAC apps that were evaluated in both the 2015 and 2017 reviews claimed to support hearing loss by offering volume control and input for listening devices (eg, earbuds). In addition, some AAC apps provided an option to adjust the speech rate, to customize the voice output, or to use a speech-to-text function. Although these features can enhance one’s listening experience, the technical specifications are not capable of being adapted to different hearing loss profiles. Therefore, future apps found in the iOS marketplace should be designed to interface with hearing aid apps (eg, Mobile Ears) running on mobile devices [41]. The significance of meeting the hearing health needs of elderly residents in LTRC is apparent when considering that most residents in LTRC are living with hearing loss [42] and that failing to accommodate to their hearing loss can have repercussions on their cognitive and social well-being [2,43,44]. For example, Amieva et al [45] reported that people living with hearing loss who use hearing aids or other assisted listening devices are much less likely to experience cognitive decline than those who do not use hearing supportive devices. These authors also provided evidence that ensuring persons with hearing loss use their hearing aids is an important factor in the person’s likelihood of using new technologies (eg, smartphone). Given that hearing aid use enables persons to engage in communication, it would follow that the use of other types of communication enhancement devices, such as cApps with features that support hearing, could be used in conjunction with hearing aids to help maintain cognitive and social functioning in aging and dementia. Future research is needed to explore the potential long-term benefits to cognitive and social health associated with regular use of hearing aids (or other assistive listening devices) and cApps in LTRC.

Many older adults in LTRC also experience significant declines in their vision [46]. This challenge can be addressed to some extent by ensuring residents are wearing appropriate corrective lenses and that the size of the images and text fonts is enough for each resident’s vision needs. However, because MCT devices are small, the upper range of expanding images and text is highly constrained by the size of the device. Consequently, there is a need for accommodating the visual needs of residents while maintaining portability. One possible solution yet to be realized would be to pair the MCT device (eg, tablet) that care staff use with special glasses for the resident that connect wirelessly to the MCT, allowing the image or text to be projected up close [47]. Another option, also yet to appear on the market, would be to use an MCT device that has an easy-to-use expandable/retractable display.

Other potential obstacles to overcome in using cApps effectively in LTRC relate to constraints on care staff in employing cApps during ADLs and on residents’ physical abilities to interact via an MCT device. First, the demands on staff attending to multiple residents within a short period would require that the cApps be easily accessed in terms of activating a resident’s customized cApp profile. This would entail having an *umbrella* home page that links to each resident’s profile, a function that is currently not available on any cApps. Second, the staff are often engaged in care activities that require them to use both arms and hands, making it difficult to switch between care tasks and the use of an MCT device. Staff would need to plan their care activities in such a way that accessing the cApp does not interfere with the task or risk injury to either them or the resident. A related constraint is that care activities require staff to be very mobile, frequently bending over, while they assist residents during ADLs. These demands would make it necessary for the MCT device to be as small as possible so that it could be positioned in an easily accessed, yet secure, pocket/holster. As mentioned above, the size of an MCT device limits the size of images and text appearing on the cApp. This double-edged challenge of portability and resident user feasibility will require creative technical and functional solutions. As Reis et al [48] note, “technologies should complement and enhance service delivery and never impose themselves as an extra burden on already work-overloaded health professionals” [48]. Other challenges for successful use of cApps in LTRC include the need for care staff to have access to Wi-Fi, to be able to seamlessly update and transfer customized settings across different care staffs’ MCT devices, and to be provided with ongoing training on how to effectively use MCT in a person-centered way during activities that are often physically and emotionally demanding.

From the resident’s perspective, the use of technology for the current generation of residents is usually a novel experience and one that may be confusing and/or unappealing to them [49]. For this reason, it would be important to introduce cApps and MCT in a gradual fashion, perhaps beginning with a minimally demanding app such as passively listening to music [50,51]. Once a resident gets accustomed to the device, a caregiver can try out additional features and functions based on the resident’s needs, abilities, and preferences. A second, and related, constraint for residents’ use of cApps is their limited capacity to point to, touch, or drag/swipe because of their lack of familiarity with a cApp interface as well as their diminished fine motor skills and tactile sensitivities (see Armstrong et al [29] for a detailed discussion). Manufacturers of MCT devices and cApps should consider how the user interface could be more suitably adapted to accommodate older adults’ motor and sensory capacities.

### Limitations

Although this study is the first to systematically search the app marketplace to identify and evaluate AAC and translation apps that would be suitable for use in the LTRC setting to support caregiver-resident communication, the review was limited to cApps found in the Canadian (English) iOS marketplace. Therefore, future research is needed to systematically review cApps available in additional platforms and app stores (eg, Google Play). To better understand the ways that cApps may change in the marketplace over time, we compared two time points: 2015 and 2017. Therefore, the percent changes reported in this study cannot be interpreted as trends in the marketplace. Finally, given the fast-changing landscape of the mobile app marketplace, future research should consider performing an app store search to verify the continued availability of the top recommended apps reported in this study.

### Future Directions

To date, there is limited empirical research published on the use of mobile technology to support caregiver-resident communication in LTRC, and there is no available evidence to support the use of any of the identified cApps for caregiver-resident communication. Therefore, there is a need for future research to empirically examine the feasibility of using currently available cApps in the LTRC setting, as well as identify gaps in the use of this technology within different LTRC contexts. A better understanding of how care staff could use cApps to support PCC in LTRC should lead to improved quality of care and quality of life for residents living in LTRC homes.

### Conclusions

The use of cApps may offer an innovative solution to support person-centered health care for residents living in LTRC homes. This study identified several cApps available in the App Store that aim to facilitate adult communication in general; however, very few cApps were designed with built-in features and custom features that would effectively support PCC in the LTRC setting. Although comparisons of our top-rated cApps demonstrated the inclusion of features that are potentially useful for supporting PCC, there was no indication that the currently available cApps were specifically designed for use in the LTRC setting to enhance caregiver-resident communication during ADLs. Furthermore, no cApp developer appeared to involve stakeholders (eg, clinicians, researchers, residents, and care staff) in the development and design process.

The ubiquitous nature of MCT (tablets/smartphones and their apps) and the growing use of mobile health in a variety of health care settings offer nurses and residential care aides an accessible and innovative tool to promote social participation and person-centered care. However, it is important to identify the availability and stability of commercially available cApps, as well as to conduct comprehensive reviews of the content and quality of existing apps, to ensure that cApps can be used to overcome communication barriers in the LTRC setting. Moreover, to improve the content and quality of cApps and to maximize the benefits of using mobile technology in care practices, it is imperative to include nurses and other care staff in the future development and design of cApps used in LTRC.

## Appendix

Multimedia Appendix 1 Screenshots of the top-recommended communication apps reviewed in 2015 and 2017.

## Abbreviations

AAC: augmentative and alternative communication
ADL: activities of daily living
ADRD: Alzheimer disease and related dementias
cApp: communication app
iOS: iPhone Operating System
LTRC: long-term residential care
MCT: mobile communication technology
PCC: person-centered communication
SLP: speech-language pathologist
