# Supplementary material for: Mobile Apps to Support Caregiver-Resident Communication in Long-Term Care: Systematic Search and Content Analysis
Source: JMIR Aging. 2020 Apr 8;3(1):e17136. doi: 10.2196/17136 (PMC7177427; doi:10.2196/17136)
Supplement: Multimedia Appendix 1 [file aging_v3i1e17136_app1.pdf]

Multimedia Appendix 1

Screenshots of the top-recommended communication apps (cApps) reviewed in 2015 and 2017.
Current URLs are provided for the cApps that are still available in the marketplace.

2015 review: recommended cApps	Screenshot (iPad)
CommuncoTool Adult	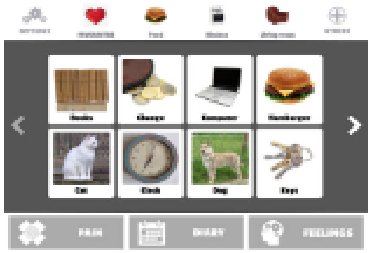
GoTalk NOW	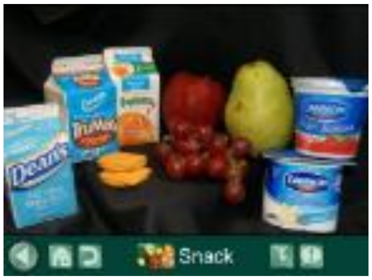
Google Translate	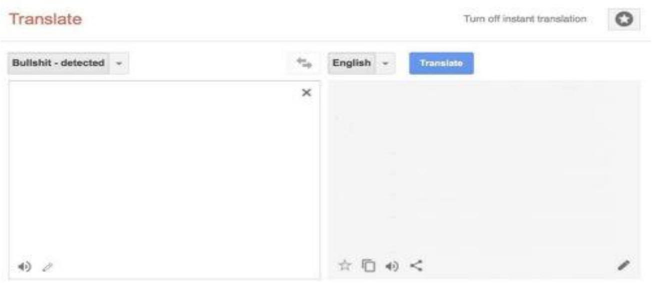
TableTop Translator<br><br>https://appadvice.com/app/tabletop-translator/576422798	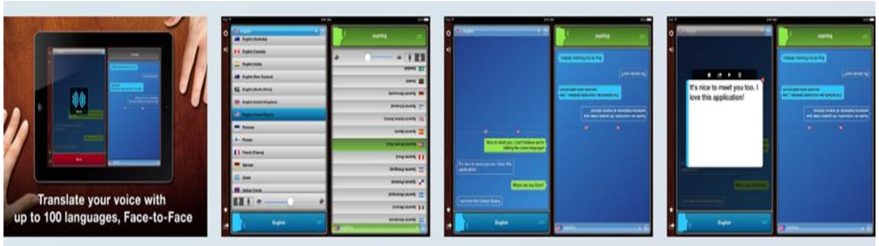

**2017 review: recommended cApps**	**Screenshot (iPad)**
CommunicoTool2<br><br>https://appadvice.com/app/communicotool-2/1034318912<br><br>https://www.amazon.ca/C-Texdev-CommunicoTool-2/dp/B01CJOIXB2	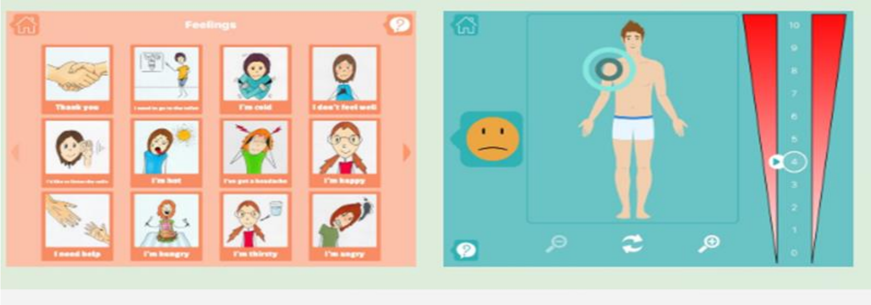
GoTalk NOW Lite<br><br>www.attainmentcompany.com<br><br>https://apps.apple.com/ca/app/gotalk-now-lite/id953164338	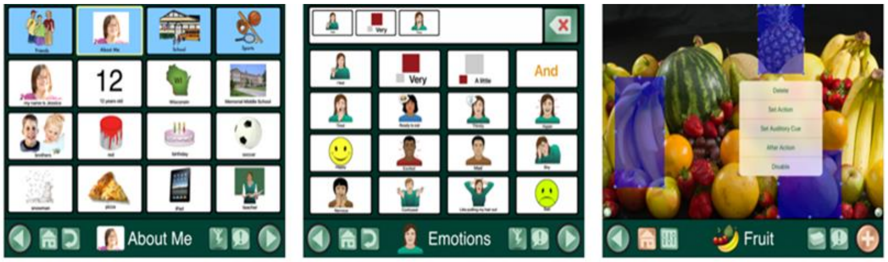
Google Translate<br><br>https://apps.apple.com/ca/app/google-translate/id414706506#?platform=ipad	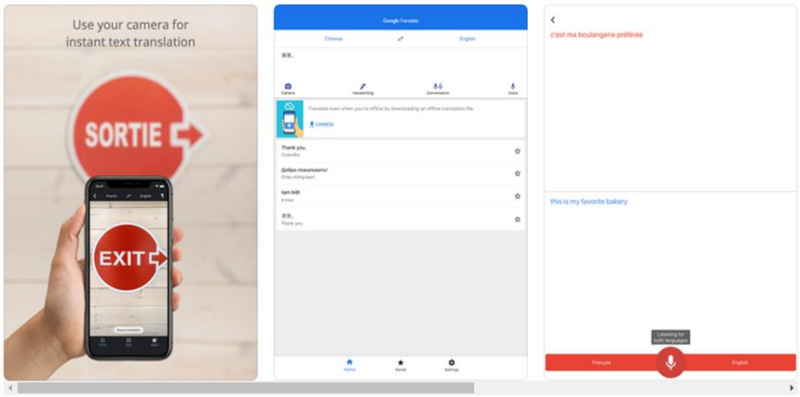
Microsoft Translate<br><br>https://apps.apple.com/ca/app/microsoft-translator/id1018949559	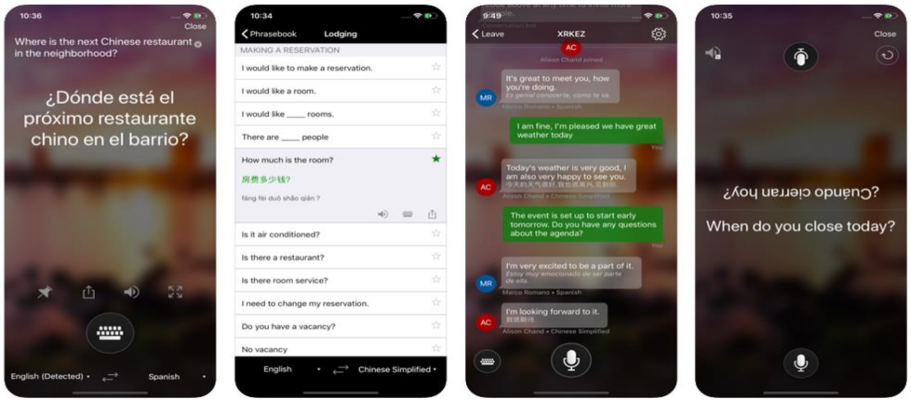
